# On-Farm Diversity and Market Participation Are Positively Associated with Dietary Diversity of Rural Mothers in Southern Benin, West Africa

**DOI:** 10.1371/journal.pone.0162535

**Published:** 2016-09-08

**Authors:** Mauricio R. Bellon, Gervais D. Ntandou-Bouzitou, Francesco Caracciolo

**Affiliations:** 1 Bioversity International, Maccarese, Italy; 2 Bioversity International, Cotonou, Benin; 3 University of Naples Federico II, Portici, Italy; TNO, NETHERLANDS

## Abstract

**Objective:**

The objective of this study is to test the extent to which, under different opportunities for market participation, the diversity of plant species rural households grow or collect (on-farm diversity), and the variety of foods mothers purchase (market diversity) are associated with their dietary diversity.

**Methods:**

Rural households from three districts in southern Benin were interviewed during dry (n = 472) and wet (n = 482) seasons between 2011 and 2012. Villages within districts and their households were selected randomly according to market accessibility, with a mother selected from each household. Information on on-farm diversity was collected using a semi-structured questionnaire. Market diversity was obtained through a 7-day food frequency questionnaire that elicited if foods were purchased. Dietary diversity was derived from a quantitative 24-hour food recall. A system of three simultaneous equations via a Generalized Methods of Moments was estimated to address potential endogeneity between dietary diversity and on-farm diversity and market diversity.

**Results:**

Results show rich on-farm diversity with more than 65 different edible plant species grown or collected by households. More than 70% of foods consumed by mothers were purchased in 55 market places. More than 50% of mothers met minimum dietary diversity with at least 5 food groups consumed. Diagnostic tests indicated the existence of endogeneity. Econometric results showed that on-farm and market diversities were positively associated with mothers’ dietary diversity (p < 0.05) once market opportunities, seasonality and other socioeconomic factors were controlled for.

**Conclusion:**

Results provide evidence of a positive relationship between on-farm diversity and dietary diversity among participant mothers. They demonstrate the important contribution of market diversity to their dietary diversity. Links among these three facets of diversity suggest that production for self-consumption and food purchases complement rather than replace each other in their contribution to dietary diversity and thus dietary quality.

## Introduction

Despite increasing homogenization of the crop species that contribute to global food supplies [1], at a local level millions of rural households (HHs) throughout the developing world continue to rely on a diversity of plant species to feed themselves and to support their livelihoods. Rural HHs manage portfolios of plant species that they cultivate or gather—referred to here as on-farm diversity (OFD)—and their specific combinations can directly affect HH nutrition and health outcomes [2]. This relationship goes beyond simply covering the calorie requirements of HH members, to influencing their dietary quality and nutritional status [2, 3]. Dietary diversity (DD) is universally recognized as a key component of healthy diets and is strongly associated with nutrient adequacy [4, 5]. Indices of DD are considered good, simple, reliable indicators of nutritional quality and adequacy [5, 6, 7, 8]. Surprisingly, very few studies have examined the link between OFD and DD in the developing world [9, 10, 11, 12]. This link may be relatively simple in highly autarkic HHs [2]. It can become, however, quite complex under increasing opportunities for market participation, where HHs have more opportunities to trade, generate income, and purchase different types of foods [10].

**Fig 1:**
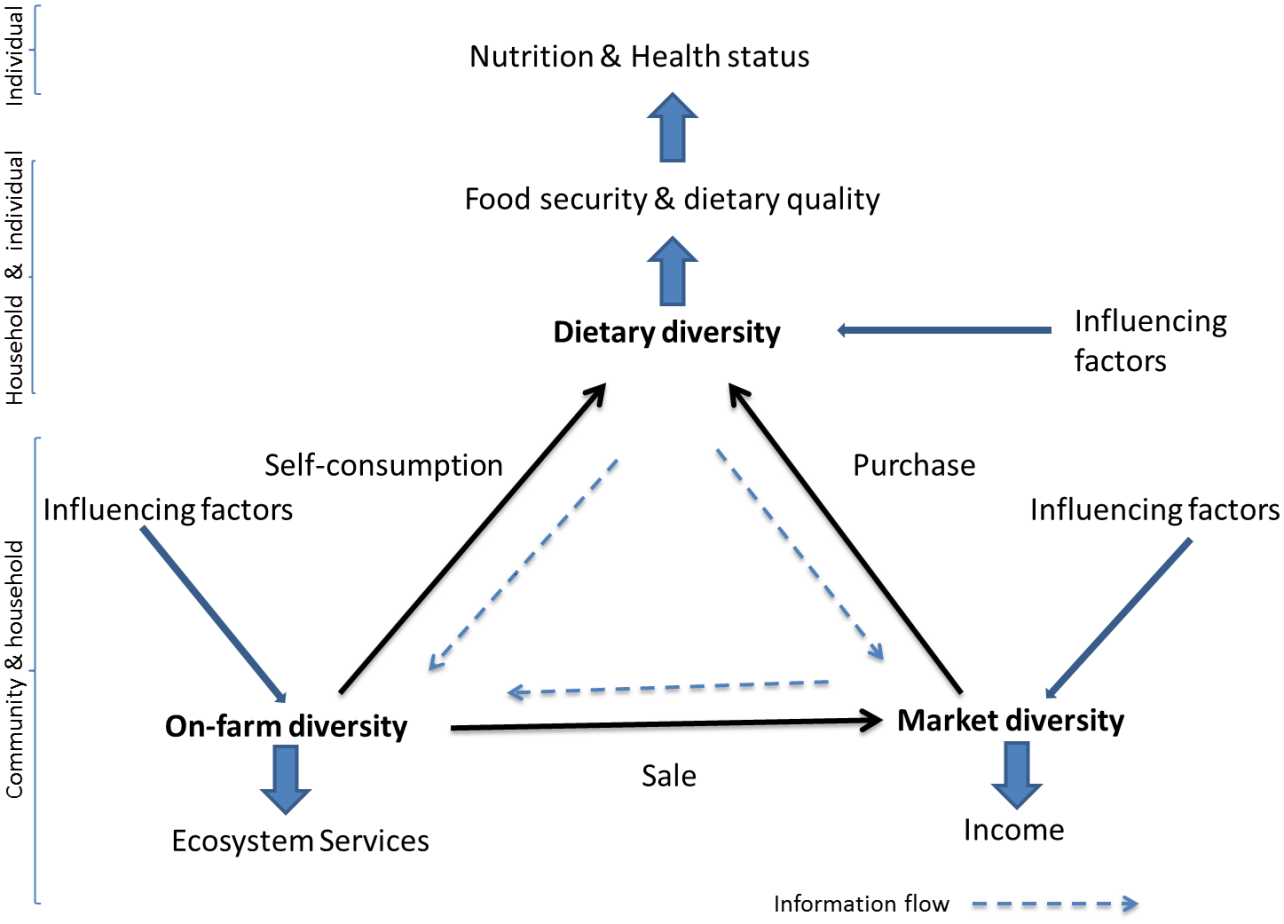
Conceptual model of the relationships among on-farm, dietary and market diversities.

Increased access and participation in markets present great opportunities, but also challenges, for rural societies [13, 14]. In rural areas of the developing world, markets often function poorly with high transaction costs; this limits market participation and fosters self-consumption [15, 16, 17, 18, 19]. Under these circumstances, HH decisions on production and consumption are not separable [19, 20] and so agricultural production influences HHs’ well-being not only directly through the income generated but also through the combination of crops produced [2]. Markets can also offer HHs opportunities to benefit from growing a diverse portfolio of species or varieties by providing opportunities to exploit seasonality and fill particular market niches [21, 22, 23]. So rural HHs´decisions about the mix of agricultural species—and associated products—to produce, consume, sell and purchase have important implications for their nutrition, but can entail great complexity. This complexity can be represented in a simple conceptual model by means of a triangle of connections among three facets of diversity (Fig 1): (a) the diversity of plants produced or gathered (OFD), (b) the diversity of foods consumed in diets (DD), and (c) the diversity of foods and products sold and purchased in markets (MD). Physical flows connect OFD and DD through self-consumption, OFD and MD through sale, and DD and MD through purchase. These flows span three different scales from the community to the HH and to the individual. There are also information flows (dash arrows) that create feedback loops (e.g. through demand, supply, prices, preferences, knowledge and tradition) across time and scales. Associated with each type of diversity, communities, HHs or individuals generate outcomes that are important for them and for society, such as food security and dietary quality, income, and ecosystem services. Each of these types of diversity is influenced by sets of exogenous factors, e.g. population density, links to different types of markets, availability of infrastructure, climatic variability, land quality and heterogeneity, land tenure, gender relationships, ethnicity, etc., within particular environmental, institutional and historic contexts. Some of these factors may influence all three types of diversity, while others may be specific to a subset. The model hypothesizes that the relationships among these three facets of diversity are endogenous, i.e. they influence each other, and thus causality among them is ambiguous. This conceptual model provides a framework to study these relationships systematically in specific contexts.

The conventional narrative of agricultural development postulates that with improved access and participation in markets, HHs’ decisions on production and consumption will uncouple. Farmers will tend to specialize in the crops where they have a comparative advantage, due to more opportunities to sell agricultural products, while purchasing the foods they consume [15], particularly since markets provide more diverse foods than any individual HH can produce. Thus under improved market access OFD should decrease as farming HHs specialize in the most profitable and marketable species; the link between OFD and DD in our model disappears and only the link between MD and DD remains relevant. This sequence of events represents the theoretical basis that inspires our research questions: (i) to what extent do varying opportunities for market participation influence the portfolio of species domesticated, semi-domesticated and wild—that rural HHs grow or collect (OFD)? (ii) do these opportunities influence the variety of foods that women of reproductive age purchase (MD)?; and (iii) how does the OFD a HH maintains and the variety of foods these women purchase influence their DD? Empirically, this study tests the hypotheses that with increased opportunities for market participation for a HH: (1) OFD decreases because of increased specialization in the most marketable and profitable crops; while (2) the variety of foods that women of reproductive age purchase increases because more foods are available and affordable; and so (3) any effect of OFD on DD disappears because of increased incentives to specialize crop production and a decreasing need to self-produce the variety of foods consumed, in turn leading to MD to be the sole factor that influences DD.

Examining how improved market access and more opportunities for rural HHs to participate in markets both as consumers and producers influence these relationships merits focused and contextual research. Reality likely diverges from theoretical expectations, being generally more complex and messy, especially in developing countries [15]. Most of the few available studies have examined the link between OFD and DD—one side of the triangle—by using simple correlation or regression analyses that show a positive association between these two factors, even after correcting for other potential confounding factors such as wealth, education, farm size, family size and gender [3, 9, 10, 11, 12, 24, 25, 26, 27, 28]. Results are context specific [10] and can be controversial [29, 30, 31]. Many of these studies however, fail to recognize the complexity of the decision-making encompassed in this relationship and the feedback loops involved, which may make the relationship between OFD and DD endogenous, and thus causality difficult to ascertain, as hypothesized in our conceptual model (Fig 1). They also ignore or treat in a very basic way the role of the diversity of foods supplied by markets (MD), the other side of the triangle, and of market participation as a key driver in these relationships. The relationships among OFD, DD and MD at the HH level have important policy implications. There are increasing calls and initiatives to promote OFD for self-consumption at farm level as a way to improve DD [9, 10, 32, 33]. Some favor however an approach which seeks improvements in DD through purchases, by increasing income generation through specialization and better productivity, and improved market participation [3, 10, 12].

These relationships are contextual, and examining them is important in the situations of low income countries, with a large share of rural population, high reliance by farmers on their own production and high incidence of undernourishment. In these cases, influencing these relationships positively can lead to important improvements for rural populations and to better pathways for the transformation of local food systems [34]. The present study focuses on Benin, a low-income country in West Africa with a high share of rural population (61%) [35]; among the rural population, about 40% of HHs are food insecure [36] and persistent chronic malnutrition (stunting) affects 15% of children under 5 years age [37]. Rural HHs that depend on agriculture, obtain between 30–36% of their food from their own production [36,38]. Local diets are dominated by staple foods (maize, rice and cassava) consumed with small amounts of vegetables, legumes and fish [39,40]. At the HH level, our study centers on women of reproductive age (mothers) since they are responsible for preparing meals and rearing children and as such their decisions affect the whole HH [7, 41, 42]. They are also some of the most nutritionally vulnerable members of rural HHs [43] and for whom there is very little information on dietary quality [8]. Biases in intra-household allocation of food have been well documented [44], and may heighten their vulnerability. For example differences in micronutrient intakes of men and women have been recognized in Sub-Saharan Africa [43]. Furthermore there is emerging evidence that women empowerment has implications on how OFD relates to their own and their children’s diets [27].

We designed a study that explicitly takes into account varying opportunities for market participation among rural HHs by selecting three districts in southern Benin with different levels of urbanization and varying ratios of urban to rural population densities. Within each district, villages located at different traveling times to a reference market center were sampled. The set of villages within a district constitutes a ‘market-shed’ [45], providing different opportunities for HHs to participate in and be influenced by a network of markets. Our expectations are that being located in a market-shed with a higher ratio of urban to rural population density offers rural HHs a larger market to sell agricultural products and their labor, as well as a higher variety of products to purchase—thus more opportunities for market participation—compared to HHs located in a market-shed with only a rural population. Even within a market-shed with a particular ratio of urban to rural populations, HHs located close to the main market places face lower costs to engage in markets both as sellers and buyers (including transaction costs), which increase as traveling times to those places rise. Therefore, HHs close to the main market place should have more market opportunities (due to lower participation and transaction costs) that diminish as traveling time increases. We use “opportunities for market participation” rather than just “market access” to underline that HHs have choices. They may decide to participate or not in market transactions and what is exogenous are the levels of choices offered to them by the market networks available (the market-shed). All studied populations were rural.

## Methods

### Setting and study design

The study took place in southern Benin. Study sites were selected within a similar agro-ecological environment based on maize growing conditions [46], due to the importance of this crop for rural populations. The study took place exclusively among rural populations of three districts in the départments (2^nd^ level administrative division in Benin) of Atlantique and Zou, with different levels of urbanization: higher urban vs. rural population densities, referred to as urban (Bohicon); higher rural vs. urban population densities, referred to as semi-urban (Allada); and purely rural populations, referred to as rural (Toffo) [47, 48] (S1 Table). A multistage sampling method was used. Sample size was 476 HHs, and was determined based on the prevalence of underweight women in the two départments to which the three studied districts belong to, relying on available data from the Demographic Health Survey (DHS) [38] and using a standard formula [49] (S1 Text).

For each district, a map with bands of travel time by car to a market center based on three accessibility levels (high: ≤ 30 min, medium: 31–59 min, low: ≥ 60 min) was developed based on distance, topography, road availability and quality, using a spatial network analysis in a Geographic Information System. Sample size was allocated proportional to the number of HHs in each of the three accessibility levels relative to the total number of HHs. The number of villages to be studied within each accessibility level was obtained by dividing the number of HHs to be sampled by 15 HH/village/level, villages were then randomly selected within a level and district, so 33 villages were studied. In each village, an average of 15 HHs with a mother (15–49 years old) and a child (6–59 months old) were randomly chosen. One mother was randomly selected in each HH to represent it. To account for seasonality two rounds of surveys were undertaken: (1) dry season (January to February 2011)—food is plentiful after the main harvest; and (2) wet season (April to May 2012)—food is scarcer as stores are depleted between harvests. Although the original intent was to interview the same HHs in both seasons this was not possible due to withdrawal, migration and difficulty in locating some of them. Thus additional HHs were interviewed during the second round to complete the sample. The first survey included 472 HHs and the second 482, with 302 in common, for a total of 652 HHs over both seasons, thereby forming a pooled sample of 954 observations. After dropping observations with incomplete information, the econometric model estimated here (see below) included 880 observations. The resulting sample size achieves a power of 0.90 (with a type I error rate of 0.05 and effect size of 0.01), according to a post-hoc power analysis provided by G*Power 3.1 [50].

### Data collection and management

Socioeconomic data were collected through face-to-face interviews with mothers (helped by their husbands) using a semi-structured questionnaire. Participants were asked about all the markets they visited in the previous 15 days, their location and the frequency of their visits. An index of HH socioeconomic status (SES) based on the ownership of bicycles, motorcycles, motor vehicles, radios, TVs, and cellphones was computed for all HHs [51].

A comprehensive list of all useful plant species grown and collected by the mother and the father in a HH during the previous agricultural season was elicited through interviews. Counts of plant species grown and collected were used as indicators of OFD at the HH level.

Qualitative food consumption data of the mother were documented using a structured food frequency questionnaire over a 7-day recall period. Data on all food items consumed were recorded in a completely disaggregated way, including different preparations of the same species, and the origin of the foods consumed: self-production, purchase, gathering, or gift. Quantitative data on foods consumed were collected from a 24-hour food recall of the mother using standard procedures [52]. Participants were asked to report all the food and drinks consumed during the preceding day regardless of place of consumption. Portion sizes and quantities of ingredients used to prepare dishes were carefully determined by standardized HH units. Detailed descriptions of the foods and mode of preparation were also collected [52].

Data on food consumed were grouped into 10 food groups in order to compute the dietary diversity score for women (WDD) [53]. Consumption of at least 15g of each group was computed and assigned a score of “1” while the consumption below 15g was scored “0.” Consumption at or above this level is more strongly associated with micronutrient adequacy for women [53]. The WDD was computed summing the food groups consumed by each individual (minimum = 0; maximum = 10). The Minimum Dietary Diversity–Women (MDD-W) a binary indicator defined as “1” if a woman consumed a minimum of 5 of the 10 food groups considered and “0” if not, was also computed [53].

### Data analysis and econometric approach

The studied decision-making process involves three main choices of the HH: consumption, production and purchase, which are interdependent and need to be evaluated simultaneously in a unified framework based on our conceptual model (Fig 1). We estimated a system of three simultaneous equations via a Generalized Method of Moments [54, 55] formulated as follows for the *i-*th HH and associated mother:

$$DD_{i}=\text{exp}\left(x_{i}^{\prime }\omega +\delta OFD_{i}+\zeta MD_{i}\right)+e_{i}$$ (1)

$$MD_{i}=x_{i}^{\prime }\lambda +z_{i}^{\prime }\beta +u_{i}$$ (2)

$$OFD_{i}=\text{exp}\left(x_{i}^{\prime }\theta +z_{i}^{\prime }\gamma \right)+v_{i}$$ (3)

where **x**_i_ is a vector of confounding factors and exogenous variables that could influence the 3 outcomes *DD*, *MD* and *OFD*. *DD* and *MD* were measured at the mother level, while *OFD* was at the HH-level. For *DD* and *OFD* a Poisson-based specification was adopted since they are measured as counts [56]; **ω**, **λ** and **θ** are the parameter vectors of the equations’ system, measuring the effects of the exogenous variables on our outcomes; **z**_i_ is a vector of instrumental variables needed to account for the potential endogeneity of *MD* and *OFD*, where ***β*** and ***γ*** are parameters of the impact of the instrumental variables on the two endogenous variables and *δ* and ζ, are parameters that measure the impact of *MD* and *OFD* on *DD*; while *e*_*i*_, *u*_*i*_ and *v*_*i*_ are the error components. Using moment conditions provide results that are asymptotically equivalent to those resulting from a full information instrumental variables efficient (FIVE) estimator [57]. This approach controls for simultaneity or reverse causality between the outcomes with the support of appropriate instrumental variables [58]. The choice of instruments should be guided both by analytical considerations and empirical findings [59], so an instrument should influence the specific outcome while being completely exogenous to the other dependent variable. Instruments included size of landholdings and the index of socioeconomic status, as a wealth indicator. Under traditional land tenure systems—where market transactions are limited or non-existent (purchase and rental of land) for the most part land size is fixed and land fragmentation is common—larger landholdings entail that farmers have to manage more plots with different soils and topographies. These farmers may, therefore, face more heterogeneous production environments and need to match their crops or varieties to the appropriate conditions to optimize production [60] thus creating an exogenous incentive to maintain OFD that is independent of consumption decisions. However farmers with larger landholdings may also produce larger surpluses and generate more income that then can be used to purchase different foods. Therefore landholding size can be used as an instrument affecting DD only indirectly through the effect of OFD on DD, but only if we also control for the income derived from agriculture. To address this issue respondents were asked to score subjectively the contribution of their agricultural production to their income in a four-point scale: “very important”, “important”, “minor importance” and “not important”; which were incorporated in the model as a set of dummies. Wealthier HHs have a higher demand for, and the income and capacity to purchase, a wider variety of foods than ‘poorer’ ones. Wealth is the result of past income, savings and investments and as such is exogenous to current production and consumption decisions, so it can be used as an instrument for purchasing behavior, addressing the endogeneity between DD and MD. Wealth was operationalized in the model through the SES index. Since wealth in agricultural societies can be associated with landholdings, incorporating wealth as a covariate in the OFD equation should contribute to control for other potential effects of landholding size on DD, improving its performance as an instrument. An additional issue is the potential endogeneity between OFD and MD. While there may be an interdependence between production (OFD) and purchasing (MD) decisions, i.e. a HH may choose levels of MD based on their level of OFD and they may choose their OFD based on what is and is not available in the market, these relations are always mediated by the consumption decision (DD) given that under high transaction costs production and consumption decisions are not made independently, i.e. the influence of OFD on MD (and vice-versa) passes-through DD (consumption decision). So by taking into account the endogeneity between OFD and DD and MD and DD, the interdependence between OFD and MD is addressed as well. Diagnostic tests were carried out to determine the existence of endogeneity and to assess the validity of the instruments: Durbin–Wu–Hausman test for endogeneity, Sargan–Hansen test of overidentifying restrictions and the Weak Instrument test [61]. It should be stressed however, that while the variables we use as instruments are predetermined and we have argued for their exogeneity, there are neither statistical tests nor narrative justification to entirely insure that they are uncorrelated with all unobservable determinants of the final outcome, as good instrumental variables should be [62]. Therefore a skeptical reader may question whether the relationships we explore are really causal. A conservative view of our results then will only claim correlations and associations in these relationships.

The confounding and exogenous variables used in the econometric model include: (a) factors that influence the opportunities for and actual participation of HHs in markets, (b) socioeconomic characteristics of the HHs and the mothers, and (c) climatic variables that may influence agricultural production (Table 1). The specific variables comprise the location of a HH in each of the market-sheds and traveling time from reference market places; given that market access and opportunities may decrease as traveling time increases but at a decreasing rate, we include a quadratic term. Since participation in labor and agricultural markets and control over their proceeds may be gender-specific, these variables are disaggregated by gender. Specific characteristics of a mother can be expected to influence outcomes: age as an indicator of experience and local knowledge, formal education as an indicator of capacity to interact with others and participate in markets, and ethnicity that may establish a cultural framework for decision-making. Family size should be important in agricultural decision-making if consumption and production decisions are linked. Given that these are rainfed areas, climate variability—both spatially and temporal—should influence agricultural production and seasonality [63]. All statistical analyses were performed using STATA software (Version 12.1, http://www.stata.com). Data available at https://dataverse.harvard.edu/dataset.xhtml?persistentId=doi%3A10.7910%2FDVN%2FU3MS5K.

**Table 1:** Definition of variables used in the econometric model.

Variable names	Variable definitions
**Dependent variables**	
On-farm diversity	Number of cultivated, wild & semi-wild species grown and collected by a HH (mother and father)
Market diversity (Purchases)	Number of different purchased food items consumed by a mother in the HH from a 7-day recall
Dietary diversity	Number of food groups (out of 10) consumed by mother in the HH by at least 15g from a 24 hour recall
**Independent variables**	
Landholdings	HH landholdings (ha)
Square of landholdings	Squared of HH landholdings (ha^2^)
Socioeconomic Index	Index of socioeconomic status, predicted 1^st^ factor from a Factor Analysis performed on the number of bicycles, motorcycles, motor vehicles, radios, TVs, and cell phones owned by a HH, calculated for all HHs in both seasons together
Urban market-shed	Dummy for the location of the HH in the urban market-shed (1 = yes, 0 = no)
Semi-urban market-shed	Dummy for the location of the HH in the semi-urban market-shed (1 = yes, 0 = no) (rural market-shed excluded)
Travel time	Travel time to main market town in the relevant market-shed (minutes)
Square of travel time	Square of the travel time (minutes^2^)
No. non-agricultural income sources –Father	Number of sources of income besides own agriculture of father, indicator of participation in non-agricultural economy
Agriculture rated very important income source-Father	Dummy of whether agricultural sources of income were rated as very important by father (1 = yes, 0 = no)
Agriculture rated important income source-Father	Dummy of whether agricultural sources of income were rated as important by father (1 = yes, 0 = no).
No. non-agricultural income sources –Mother	Number of sources of income besides own agriculture of mother, indicator of participation in non-agricultural economy
Agriculture rated very important income source-Mother	Dummy of whether agricultural sources of income were rated as very important by mother (1 = yes, 0 = no)
Agriculture rated important income source-Mother	Dummy of whether agricultural sources of income were rated as important by mother (1 = yes, 0 = no).
Mother age	Age of mother (years)
Mother education	Number of years of formal schooling completed by mother
Mother ethnicity	Ethnicity of mother (1 = Aizo, 0 = else)
Family size	Family size, number of family members
Temperature range^1^	Temperature range (°C)
Coefficient of variation precipitation^1^	Precipitation Seasonality (Coefficient of Variation)
Precipitation range^1^	Precipitation range (highest and lowest rainfall during a year, mm)
Season	1 = dry season—harvesting season, January to February 2011; 0 = rainy season-planting season, April to May 2012

^1^Climatic data extracted from WorldClim (www.worldclim.org) for villages based on their geolocation (interpolations of observed data, representative of 1950–2000).

^2^ Refers to the square of the unit used.

### Ethical considerations

The study was approved by the National Ethics Committee of Benin. The subjects, as well as heads of selected households and local authorities, were informed of the purpose and procedures of the study. All participants signed an informed consent form and were enrolled in the study on a voluntary basis.

## Results

### HH characteristics

HHs tended to be young with low levels of formal education, farming small landholdings, but several were landless (Table 2). Agriculture was rated as an important income source by fathers, but much less so by mothers. Non-agricultural income sources were common among both. Few HHs had members who had migrated. HHs visited between 36 and 42 market places in a 15 day period during the dry and wet seasons respectively (55 in total). On average, each HH visited more than one market place. Very few did not visit any at all. Mean traveling times from a village to the reference market place in each market-shed showed little variation. In general, HHs operate in a typical rural economy, with small-scale agriculture as the mainstay of livelihoods, but interacting with other sectors of the economy and with wide participation in markets.

**Table 2:** HH characteristics^1^.

			Dry season 2011								Wet season 2012					
Indicator by HH	Urban		Semi-urban		Rural		Total		Urban		Semi-urban		Rural		Total	
No. of villages	9		11		13		33		9		11		13		33	
No. of HHs	114		166		130		410		115		176		191		482	
Mean age of father (years)	40.2	(9.6)	36.7	(7.9)	37.4	(9.7)	37.9	(9.1)	41.6	(9.4)	39.1	(10.7)	37.1	(7.7)	38.9	(9.4)
Mean education of father (years)	4.5	(4.2)	4.1	(4.0)	4.1	(3.7)	4.2	(4.0)	4.5	(4.2)	4.8	(4.5)	4.3	(3.8)	4.5	(4.1)
Mean age of mother (years)	30.0	(6.5)	28.8	(5.6)	28.7	(6.0)	29.1	(6.0)	31.2	(6.4)	29.7	(6.3)	29.3	(6.2)	29.9	(6.3)
Mean education of mother (years)	1.4	(2.9)	1.2	(2.4)	1.1	(2.3)	1.2	(2.5)	1.1	(2.6)	1.5	(2.7)	1.0	(2.1)	1.2	(2.5)
Mean family size (number)	7.3	(3.0)	6.3	(3.0)	5.6	(1.7)	6.4	(2.7)	6.7	(2.2)	5.6	(1.9)	5.7	(1.9)	5.9	(2.1)
Mean landholding (ha)^2^	3.1	(3.4)	2.3	(2.7)	1.4	(1.4)	2.2	(2.7)	2.5	(2.5)	1.5	(1.3)	1.0	(1.3)	1.5	(1.7)
Mean no. of sources of income outside own agriculture father	1.1	(0.8)	1.1	(0.8)	0.7	(0.5)	1.0	(0.8)	1.1	(0.6)	1.0	(0.8)	0.9	(0.6)	1.0	(0.7)
Mean no. of sources of income outside own agriculture mother	1.6	(0.7)	2.3	(0.6)	0.8	(0.5)	1.6	(0.9)	1.1	(0.6)	1.4	(0.8)	1.2	(0.7)	1.3	(0.7)
Landless HHs (%)	33.3		31.1		35.4		33.1		20.9		12.5		13.6		14.9	
Agriculture important source of income fathers (%)	55.3		67.7		47.7		57.9		67.5		67.6		67.5		67.6	
Agriculture important source of income mothers (%)	28.1		34.7		12.3		25.8		48.7		54.6		42.9		48.6	
HH with migrants (%)	2.6		1.2		14.6		5.9		1.7		9.1		3.1		5.0	
Mean travel time to reference marketplace within market-shed (minutes)^3^	30.0	(25.5)	31.3	(14.7)	32.1	(28.1)	31.3	(23.3)	30.0	(25.6)	31.6	(14.6)	31.0	(28.3)	31.0	(23.5)
No. of markets visited by HHs in 15 day period^4^	14		16		12		42		15		12		9		36	
Mean no. of markets visited by a HH in 15 day period	1.2	(0.6)	1.6	(0.6)	1.3	(0.7)	1.4	(0.7)	1.4	(0.7)	1.6	(0.7)	1.3	(0.6)	1.4	(0.7)
No. of HHs not visiting any market in 15 day period	0		2		3		5		1		1		1		3	

^1^Standard deviations in parentheses

^2^Exclude landless HHs

^3^The reference marketplaces for the market-sheds are: Bohicon for the urban, Allada for the semi-urban and Toffo for the rural

^4^HHs visited a total of 55 distinct market places when results of both seasons are considered together

### On-farm diversity

HHs grew and collected a total of 65 species over the two seasons studied—including crops and fruit trees, and wild species, mostly trees and bushes (S2 Table). Table 3 shows that the number of species was different between seasons (a total of 42 in the dry and 61 in the wet) and also varied by market-shed. The number used in any particular village was on average about half of the species available at the market-shed level. HHs who grew or collected plants managed portfolios composed of between 3 and 9 species on average, depending on market-shed and season. The highest mean number was in the urban, followed by the semi-urban and the lowest in the rural market-shed regardless of season. Overall numbers were higher in the wet compared to the dry season. The majority of species were used for self-consumption or for self-consumption and sale; few were exclusively for sale, indicating both the importance of self-consumption, as well as the complementarity between self-consumption and sale.

**Table 3:** Diversity of plant species grown and collected by HHs (OFD)^1^.

			Dry season 2011								Wet season 2012					
Variable	Urban		Semi-urban		Rural		All		Urban		Semi-urban		Rural		All	
No. of villages	9		11		13		33		9		11		13		33	
No. of HHs	114		166		130		410		115		176		191		482	
On-Farm Diversity																
Total no. of species grown & collected by HHs^2^	34		29		18		42		41		47		40		61	
Mean no. of species grown & collected by village	17.2	(8.0)	16.7	(2.2)	7.3	(2.3)	13.2	(6.5)	21.9	(6.1)	25.2	(3.0)	21.5	(2.9)	22.8	(4.2)
Mean no. of species grown & collected by a HH^3^																
overall	6.5	(4.2)	5.4	(2.6)	3.4	(1.8)	5.1	(3.1)	9.1	(4.2)	6.9	(3.1)	5.4	(2.9)	6.8	(3.6)
exclusively for self-consumption	4.4	(3.5)	1.0	(1.4)	0.9	(1.2)	1.9	(2.5)	5.6	(2.4)	3.6	(2.2)	2.0	(2.1)	3.4	(2.6)
exclusively for sale	0.2	(0.5)	1.8	(1.8)	0.9	(1.1)	1.1	(1.5)	0.2	(0.5)	0.4	(0.8)	0.4	(0.9)	0.3	(0.8)
for self-consumption & sale	1.9	(1.8)	2.4	(1.5)	1.5	(1.6)	2.0	(1.7)	3.4	(3.5)	2.9	(2.4)	3.0	(2.8)	3.0	(2.8)

^1^Standard deviations in parentheses

^2^There were an overall total of 65 plant species when results of both seasons are considered together

^3^Means exclude those HHs that did not grow or collect any species

^4^Data on consumption are from 24-hour recall of mothers, a minimum consumption of 15g for each food group was required to count the group

### Variety of foods consumed and resulting dietary diversity

Table 4 shows that mothers consumed a total of 178 and 199 different foods over a 7 day-period during the dry and wet seasons respectively. Foods were sourced in 4 ways: self-produced, gathered, purchased, or as gifts. Purchase was the dominant source across market-sheds and seasons, accounting for between 65% and 80% of all the variety of foods consumed (i.e. MD), followed by self-production, contributing only between 13% and 21% and gathered foods between 4% to 19%. Self-production and gathered foods jointly contributed between 16% and 28% depending on market-shed and season. Food gifts accounted only for between 3% and 10% of the variation of foods consumed. These data illustrate the important role of markets as sources of a variety of foods for mothers across market-sheds and seasons.

**Table 4:** Diversity of foods consumed by mothers and their sources^1^.

			Dry season 2011								Wet season 2012					
Variable	Urban		Semi-urban		Rural		All		Urban		Semi-urban		Rural		All	
No. of villages	9		11		13		33		9		11		13		33	
No. of HHs	115		174		183		472		115		174		188		477	
Total no. of different foods consumed by mothers 7 day period	156		133		143		177		130		159		173		199	
Mean no. of different foods consumed by village	92.2	(11.6)	77.5	(7.2)	79.9	(16.5)	82.5	(13.8)	69.6	(7.9)	80.0	(12.3)	79.2	(8.3)	76.8	(10.4)
Mean no. of different foods consumed by a mother 7 day period by source																
overall	28.5	(10.7)	22.5	(8.4)	24.4	(9.7)	24.7	(9.7)	23.6	(6.6)	24.3	(8.7)	25.6	(7.1)	24.7	(7.6)
purchased^2^	20.8	(9.5)	14.7	(5.6)	16.5	(7.6)	16.9	(7.8)	16.4	(7.1)	18.8	(7.5)	20.6	(6.5)	18.9	(7.2)
self-produced	5.2	(5.1)	4.3	(2.4)	3.1	(2.4)	4.1	(3.4)	5.0	(3.6)	2.7	(2.4)	3.0	(2.8)	3.4	(3.0)
gathered	1.7	(1.9)	2.0	(1.8)	2.4	(2.2)	2.1	(2.0)	1.5	(1.7)	1.2	(1.2)	1.1	(1.2)	1.2	(1.3)
gift	0.7	(1.1)	1.4	(1.7)	2.4	(2.1)	1.6	(1.8)	0.7	(1.2)	1.7	(1.8)	0.8	(1.4)	1.1	(1.6)

^1^Standard deviations in parentheses

^2^This is what we refer to as market diversity (MD)

Table 5 presents the percentage of mothers consuming at least 15g of each of 10 food groups over the previous 24 hours, a DD score based on the number of food groups consumed and the derived minimum dietary diversity for women (MDD-W). It shows that all mothers consumed more than the threshold amount of grains, roots and tubers. However, for the other food groups there was a marked variation between seasons and among market-sheds. A high percentage of mothers consumed more than the threshold amount of meats, fish and seafood. Eggs and dairy products were consumed by very few women, however many consumed vitamin A rich leafy vegetables, particularly in the wet season. Consumption of other Vitamin A rich vegetables and fruit and other vegetables varied significantly between seasons. The average number of food groups consumed by mothers was below five across market-sheds and seasons, however in the dry season more than 60% of mothers consumed five or more food groups, although this drops substantially during the wet season in the semi-urban and rural market-sheds.

**Table 5:** Food groups consumed and dietary diversity of mothers (DD)^1^.

			Dry season 2011								Wet season 2012					
Variable	Urban		Semi-urban		Rural		All		Urban		Semi-urban		Rural		All	
No. of villages	9		11		13		33		9		11		13		33	
No. of HHs	113		170		171		454		115		176		191		482	
% of mothers who consumed a food group^2^																
Grains, roots and tubers	100		100		100		100		100		100		100		100	
Legumes	52.2		48.8		50.3		50.2		63.5		52.8		51.3		54.8	
Nuts and seed	23		43.5		35.1		35.2		33		68.8		38.7		48.3	
Milk products	0.9		2.4		1.2		1.5		4.3		2.3		1		2.3	
Meats, fish and seafood	80.5		71.2		66.7		71.8		56.5		71		64.4		64.9	
Eggs	1.8		1.8		0.6		1.3		2.6		3.4		2.6		2.9	
Vitamin A rich leafy vegetables	56.6		47.6		42.7		48		78.3		55.7		58.1		62	
Other Vitamin A rich vegetables and fruits	0		0.6		0		0.2		71.3		25		27.7		37.1	
Other vegetables	90.3		78.2		89.5		85.5		3.5		5.7		8.9		6.4	
Other fruits	78.8		78.2		87.1		81.7		64.3		36.4		62.3		53.3	
Mean DD score^3^	4.8	(1.0)	4.7	(1.3)	4.7	(1.0)	4.8	(1.1)	4.8	(0.9)	4.2	(1.3)	4.2	(1.1)	4.3	(1.1)
Minimum Dietary Diversity-Women (%)	69.9		62.9		61.4		64.1		63.5		39.2		37.7		44.4	

^1^Standard deviations in parentheses

^2^Data on consumption are from 24-hour recall of mothers, a minimum consumption of 15g for each food group was required to count the group

^3^A minimum consumption of 15g for each food group was required to be included it in the dietary diversity score

### Econometric model

Diagnostic tests indicate the existence of endogeneity (Durbin–Wu–Hausman test, p<0.001, small values indicating inconsistency of Ordinary Least Squares regression), providing evidence of that HH decisions on DD are interdependent with OFD and MD. They also confirm the relevance of the instruments (Test for Weak Instruments, p < 0.001, small p-values indicate instrument relevance) and their appropriateness to address endogeneity (Sargan–Hansen test of overidentifying restrictions, p = 0.364, small p-values indicate instrument inconsistency).

Table 6 shows that OFD at the HH level and MD purchased by mothers had a positive statistically significant association with mothers’ DD once we corrected for endogeneity, opportunities for market participation and other confounding variables. This suggests that hypothesis 3, i.e. the effect of OFD on DD disappears because of crop production specialization, should be rejected. It could be argued however that since we used dummies to control for the effect of the market-shed, the results only show a weighted average of the effect of OFD on DD across market-sheds. For instance, the results cannot exclude that the effect of OFD was significant in the rural market-shed, but not in the urban, as hypothesis 3 predicts. To address this issue we included an interaction term between OFD and the rural market-shed in the DD equation (S3 Table), although this approach is not without limitations [3]. The interaction term was not statistically significant while OFD continue to be, suggesting that being located in a rural area versus an urban or semi-urban does not change significantly the direction and magnitude of this relation.

**Table 6:** Results of a system of simultaneous equations^1^modeling OFD, MD and DD as outcome variables.

Variable	On-Farm Diversity		Market Diversity		Dietary Diversity	
Constant	6.592	*	17.865		2.898	
On-Farm Diversity					0.036	*
Market Diversity					0.023	*
Landholdings	0.11	***	-0.315			
Square of landholdings	-0.005	*	0.002			
Socioeconomic Index	-0.017		1.148	**		
Urban market-shed	0.543	***	-0.794		0.093	
Semi-urban market-shed	-0.083		-2.653	*	-0.023	
Travel time	0.016	***	-0.196	***	0.007	
Square of travel time	-0.0002	***	0.001	**	-0.0006	
No. non-agricultural income sources father	0.018		0.285		-0.013	
Agriculture rated very important income source father	0.5	***	-0.27		-0.054	
Agriculture rated important income source father	0.485	***	-0.225		-0.08	
No. non-agricultural income sources mother	0.153	***	0.225		-0.025	
Agriculture rated very important income source mother	0.362	***	-2.512	***	-0.009	
Agriculture rated important income source mother	0.383	***	-0.507		-0.052	
Mother age	-0.007	*	0.097	*	-0.001	
Mother education	-0.004		0.306	**	0.002	
Mother ethnicity (Aizo)	0.051		-3.026	***	0.062	
Family size	0.031	**	0.053		-0.001	
Temperature range	-1.247	*	-5.349		-0.297	
Coefficient of variation precipitation	-0.038		0.711		-0.039	
Precipitation range	0.001		-0.06		0.004	
Season (dry)	-0.516	***	-1.153	*	0.208	***

^1^N = 880. Robust standard errors clustered at the household level to account for the fact that some households are represented twice in the data. Estimates of standard-errors do not vary significantly to those reported if clustered at the village level.

Significance at the .05, .01, .001 level indicated by *, **, *** respectively for a two-tail t-test

The instrumental variables related to landholdings were related positively with OFD but not with DD; while the wealth index was associated positively with MD but not with OFD. The signs of these relationships are consistent with a priori expectations proposed for their inclusion. Concerning the hypothesis on the existence of a negative relation between OFD and opportunities for market participation (hypothesis 1), HHs in the urban market-shed maintained a higher OFD. However, OFD was also positively related to travel time to the main market, suggesting that opportunities for market participation have differing ways of influencing OFD. Sources of non-farm income of mothers and the ratings of the importance of agriculture as an income source for both fathers and mothers were statistically significant and positively related to OFD. Older mothers tended to maintain lower levels of OFD, but larger families had higher levels. Seasonal differences were highly significant. MD was inversely related to travel time, supporting the hypothesis that opportunities for market participation increase availability of more diverse and affordable foods (hypothesis 2). Moreover mothers who rated income from agriculture as very important had a lower MD compared to others. Mothers’ age, education and ethnicity, as well as the season had a significant impact on MD. DD was only associated with seasonality. Comparable results were obtained using the Minimum Dietary Diversity–Women index as a dependent variable with a similar estimation procedure, showing the robustness of our results (S4 Table).

## Discussion

Our results provide empirical evidence of the existence of a positive relationship between OFD and DD among mothers in rural HHs in southern Benin, supporting similar findings of previous studies [3, 9, 10, 12, 24, 25, 26, 27, 28]. They also demonstrate the important role that MD plays in DD and show the presence of endogeneity among production, purchasing and consumption decisions. Results show that opportunities for market participation are highly associated with the OFD maintained by HHs, but this relationship is more complex than originally thought. On the one hand, being located in the urban market-shed, and thus having more opportunities to participate in larger and differentiated markets as producer and consumer, was linked with higher levels of OFD, suggesting that increased market opportunities foster diversification rather than specialization. On the other hand however, the positive relationship between travel time and OFD suggests that transaction and opportunity costs which limit market opportunities and participation are present as well (travel time is widely used to proxy for these types of costs[16]) and foster OFD regardless of the type of market-shed where a HH is located.

Regarding our second hypotheses about the influence of market opportunities on the variety of foods purchased by mothers (MD), results show that HHs located in the semi-urban market-shed purchased a lower variety of foods than those located in the other two market-sheds. The semi-urban market-shed may combine features of both the urban and the rural market-sheds in terms of food consumption, being similar to the former for certain foods, but to the latter for others [64], thus suggesting a non-linearity in the way that opportunities for market participation relate to MD. Travel time and thus opportunity or transaction costs however are negatively associated with MD, since they lower the availability of different foods (e.g. by increasing search costs and effort to obtain them) and likely increase their price due to higher transportation costs. This effect was present regardless of market-shed. It should be pointed out that social access to food, such as gifting foods, while not part of the econometric model, was present in our study sites. It played however a relatively minor role quantitatively compared to self-production and purchases. This does not mean that it is not important for particular people or in certain circumstances, but the analysis of its role may require more qualitative methods and adds more complexity to the issues identified here, although it merits further research.

Finally, results show that both OFD and MD are positively associated with mothers’ DD, regardless of market-shed and travel time. These results, although with qualifications, suggest that our third hypothesis should be rejected. It is worth noting that seasonality was a significant factor in all three equations. These results are consistent with the relationships postulated in our conceptual model and remain valid at least under the contexts in which this research took place. The existence of endogeneity in the relationships between OFD and DD, and MD and DD, as posited, was confirmed, and the instrumental variables used to address this issue proved to be relevant and show the expected signs. Thus environmental heterogeneity and wealth are important drivers of OFD and MD respectively.

Our study is observational, similar to previous studies that have looked at these relationships [3, 9, 10, 12, 24, 25, 26, 27]; and as those that recognize endogeneity as an issue [3, 12, 27], we have used an instrumental variable approach. Even though the exogeneity of our instruments was successfully tested conditional on the control variables we included in the model, as noted earlier, we cannot completely exclude that instruments are correlated with the unobservable determinants of the final outcome. Therefore a conservative view of our results then will only claim correlations and associations in these relationships; however even in this case our results are based on a clear conceptual framework and are consistent with previous studies.

Our findings can contribute to addressing an important and broad policy question: what type of interventions are more effective at increasing DD and dietary quality for mothers and their HHs (and thus should be supported): Are they those that increase HHs’ ability to purchase different foods or those that increase their ability to produce more diverse foods for self-consumption? Some studies [3, 10], while recognizing the evidence of a positive link between OFD and DD, argue that it decreases or disappears with increased HH participation in markets. Hence, policies that foster agricultural specialization and productivity increases, and that lead to increased monetary income to purchase diverse foods, may be more appropriate in the long run than fostering diverse self-production. This argument fits well with the narrative of the need to transform subsistence farmers into market-oriented ones [14, 65].

Overall our results indicate the complementary nature of OFD and MD on mothers’ diets, and suggest that a strategy of diversification under opportunities for market participation can have important positive effects on mothers’ DD and by extension on HHs as well. This is at odds with the view that market participation fosters specialization in a few crops. Considering the whole species portfolio and consumption patterns challenges the dichotomy of subsistence versus market-oriented farmers. The relative value of self-production versus production for the market and of purchasing food for improving rural HH diets are likely context specific and will depend on the stage of development of the food system. One should not assume that specialization is the best or only path for improving the lives and livelihoods of rural HHs under the environments, market conditions and scales at which they operate.

Our results also show that the links among OFD, MD and DD are more complex than is perhaps generally appreciated. Most HHs visited at least one local market, many species were produced both for self-consumption and for sale, and HHs purchased most of the variety of food items they consumed. In local markets it is likely that the products sold by one HH are purchased by another nearby. Therefore strengthening the capacity of HHs to sell in local markets may provide opportunities to improve their consumption as well. Markets and the diversity of foods and products traded in them not only provide diversity to diets, but also support OFD by providing an outlet for HH production. In spite of the positive role of OFD and MD on DD, our results show that about a third of the mothers in the sample have low levels of DD, and that this deteriorates in the wet season in areas with less market access. This suggests the need to address seasonality in diet quality and reinforce the importance of market access and thereby MD for improving diets. Influencing the performance of local markets presents opportunities to enhance HHs’ well-being, as both consumers and producers of agricultural products. Any such efforts need to recognize the diversity of species and foods that are traded in these markets, rather than focusing just on improving the value chains of a few species. Markets are more than value chains of particular crops. The structure and dynamics of local markets, in their diversity and with the challenges they entail, need to be better understood and influenced, an area that merits further research [66]. Rather than focusing exclusively on improving specialization, productivity and markets of one or two crops, there may be important opportunities to look at how to improve the composition and performance of a portfolio of species to deliver more food, better nutrition and higher income [9, 67].

Fostering trajectories of change for rural food systems as economies develop that build on local diversity and local markets, and with a particular focus on better diets and nutrition, may be an avenue worth exploring in a systematic way. It fits well with the recognized need to move towards more diversified production systems that deliver higher dietary quality and that look at the food system in a holistic way [68, 69]. Designing and testing interventions to support diversification patterns that enhance the complementary role of species portfolios for self-consumption and as a source of income for rural HHs can be an important way to improve their diets. This requires however a perspective that goes beyond just the production side, and incorporates consumption patterns, dietary quality, and local market structures and dynamics.

## Supporting Information

S1 Table Secondary data on studied districts. (DOCX)

S2 Table Plant species grown or collected and objective of production by season. (DOCX)

S3 Table Results of a system of simultaneous equations modeling OFD, MD and DD as outcome variables including an interaction term between OFD and the rural market-shed. (DOCX)

S4 Table Results of a system of simultaneous equations modeling OFD, MD and Minimum Dietary Diversity—Women as outcome variables. (DOCX)

S1 Text (DOCX)
