# Supplementary material for: On-Farm Diversity and Market Participation Are Positively Associated with Dietary Diversity of Rural Mothers in Southern Benin, West Africa
Source: PLoS One. 2016 Sep 8;11(9):e0162535. doi: 10.1371/journal.pone.0162535 (PMC5015832; doi:10.1371/journal.pone.0162535)
Supplement: S1 Table — (DOCX) [file pone.0162535.s001.docx]

Table S1. Secondary data on studied districts^1^

|  |  |  | Market-shed |  |  |  |  |  |
| --- | --- | --- | --- | --- | --- | --- | --- | --- |
| Variable | Urban |  | Semi-urban |  | Rural |  | All |  |
| No. of villages | 9 |  | 11 |  | 13 |  | 33 |  |
| Mean rural population density (persons/ km^2^) ^2^ | 163.1 | (91.9) | 236.5 | (15.7) | 171.5 | (21.8) | 190.9 | (58.8) |
| Mean urban population density (persons/km^2^)^3^ | 480.9 | (560.5) | 26.6 | (21.4) | 0 |  | 140.0 | (351.8) |
| Mean travel time to nearest town over 20,000 inhabitants (hours, 2000)^4^ | 1.0 | (0.8) | 1.6 | (0.4) | 1.8 | (0.4) | 1.5 | (0.6) |

^1^Standard deviations in parentheses

^2^HarvestChoice, 2015. "Population Density, rural (pers./sq. km., circa 2005)." International Food Policy Research Institute, Washington, DC., and University of Minnesota, St. Paul, MN. Available online at <http://harvestchoice.org/data/pd05_rur>. These data are provided in a grid and were imported into a SIG program (DIVA-GIS). The closest grid cell to each of the communities in our sample was visually identified (within 2 and 6 km) and the value of that grid cell used. In multiple cases, several villages where located closest to the same grid cell and the corresponding value was used in all of them. The same procedure was used for the urban population and the travel distance.
