# Supplementary material for: On-Farm Diversity and Market Participation Are Positively Associated with Dietary Diversity of Rural Mothers in Southern Benin, West Africa
Source: PLoS One. 2016 Sep 8;11(9):e0162535. doi: 10.1371/journal.pone.0162535 (PMC5015832; doi:10.1371/journal.pone.0162535)
Supplement: S2 Table — (DOCX) [file pone.0162535.s002.docx]

Table S2. Plant species grown or collected and objective of production by season

|  |  | Dry season | | | | |  | Wet season | | | | |
| --- | --- | --- | --- | --- | --- | --- | --- | --- | --- | --- | --- | --- |
|  |  |  | Objective (%) | | | |  |  | Objective (%) | | | |
| Scientific name | English name | Total (N=302) | self-consump-tion | market only | both | no data |  | Total (N=440) | self-consump-tion | market only | both | no data |
| *Zea mays* L. | Maize | 97.7 | 40.1 | 3.3 | 52.0 | 2.3 |  | 90.0 | 45.0 | 2.0 | 43.0 | 0.0 |
| *Manihot esculenta* Crantz | Cassava | 75.5 | 18.9 | 16.2 | 37.7 | 2.6 |  | 86.8 | 30.7 | 5.9 | 49.8 | 0.5 |
| *Vigna unguiculata* (L.) Walp. | Cowpea | 42.7 | 16.6 | 2.0 | 22.8 | 1.3 |  | 39.1 | 16.4 | 0.9 | 21.8 | 0.0 |
| *Mangifera indica* L. | Mango | 11.9 | 10.9 | 0.0 | 0.7 | 0.3 |  | 58.6 | 44.8 | 0.7 | 12.5 | 0.7 |
| *Solanum macrocarpon* L. | African eggplant | 25.5 | 9.6 | 9.3 | 6.0 | 0.7 |  | 30.2 | 13.2 | 0.7 | 16.4 | 0.0 |
| *Lycopersicon esculentum* Mill. | Tomato | 17.9 | 2.0 | 5.6 | 9.6 | 0.7 |  | 32.7 | 3.6 | 5.2 | 23.9 | 0.0 |
| *Elaeis guineensis* Jacq. | Palm tree nut | 31.1 | 4.6 | 4.6 | 15.9 | 6.0 |  | 16.8 | 1.6 | 2.3 | 12.7 | 0.2 |
| *Vernonia amygdalina* Delile | Bitter leaves | 11.6 | 8.6 | 1.7 | 1.0 | 0.3 |  | 35.7 | 29.1 | 0.2 | 6.4 | 0.0 |
| *Corchorus olitorius* L. | Jew’s mallow | 17.9 | 8.6 | 5.3 | 3.3 | 0.7 |  | 25.2 | 13.9 | 1.6 | 9.8 | 0.0 |
| *Arachis hypogaea* L. | Peanut | 20.2 | 1.3 | 7.9 | 10.3 | 0.7 |  | 20.7 | 4.1 | 1.1 | 15.2 | 0.2 |
| *Citrus sinensis* (L.) Osbeck | Sweet orange | 6.6 | 4.6 | 0.7 | 1.3 | 0.0 |  | 31.6 | 17.5 | 1.1 | 13.0 | 0.0 |
| Musa acuminata Colla | Sweet bananas | 21.5 | 3.6 | 11.9 | 5.0 | 1.0 |  | 13.9 | 5.0 | 1.1 | 7.7 | 0.0 |
| *Capsicum annuum* L. | Pepper | 13.6 | 1.3 | 4.3 | 7.9 | 0.0 |  | 19.8 | 4.8 | 2.0 | 13.0 | 0.0 |
| *Ananas comosus* (L.) Merr. | Pineapple | 16.6 | 1.7 | 13.2 | 1.0 | 0.7 |  | 13.2 | 1.1 | 4.8 | 7.0 | 0.2 |
| *Moringa oleifera* Lam. | Drum‐stick | 5.6 | 5.3 | 0.0 | 0.3 | 0.0 |  | 22.3 | 21.6 | 0.0 | 0.7 | 0.0 |
| Musa × paradisiaca L. | Plantain | 16.9 | 0.7 | 11.3 | 4.3 | 0.7 |  | 8.0 | 0.7 | 2.0 | 5.2 | 0.0 |
| *Amaranthus cruentus L.* | Amaranth | 10.6 | 3.3 | 5.3 | 1.7 | 0.3 |  | 13.2 | 7.3 | 0.7 | 5.2 | 0.0 |
| *Abelmoschus esculentus* (L.) Moench | Okra | 10.6 | 3.6 | 5.0 | 1.7 | 0.3 |  | 11.6 | 2.7 | 0.7 | 8.2 | 0.0 |
| *Celosia argentea* L. | Cockscomb | 10.6 | 6.0 | 2.6 | 2.0 | 0.0 |  | 11.6 | 8.4 | 0.2 | 3.0 | 0.0 |
| *Carica papaya* L. | Papaw | 3.6 | 3.3 | 0.3 | 0.0 | 0.0 |  | 13.0 | 10.7 | 0.0 | 1.8 | 0.5 |
| *Vitex doniana* Sweet | Black plum | 6.0 | 6.0 | 0.0 | 0.0 | 0.0 |  | 10.5 | 9.1 | 0.0 | 1.4 | 0.0 |
| *Ipomoea batatas* (L.) Lam. | Sweet potato | 11.3 | 3.0 | 1.0 | 6.6 | 0.7 |  | 3.2 | 0.2 | 0.9 | 2.0 | 0.0 |
| Irvingia gabonensis (Aubry-Lecomte ex O'Rorke) Baill. | Wild mango | 2.0 | 1.7 | 0.0 | 0.3 | 0.0 |  | 12.3 | 9.8 | 0.0 | 2.5 | 0.0 |
| *Glycine max* (L.) Merr. | Soya bean | 6.3 | 2.3 | 2.3 | 1.7 | 0.0 |  | 7.3 | 0.7 | 0.9 | 5.7 | 0.0 |
| *Dioscorea cayennensis* Lam. | Yellow Guinea yam | 2.3 | 2.0 | 0.0 | 0.3 | 0.0 |  | 9.1 | 6.8 | 0.0 | 2.3 | 0.0 |
| *Psidium guajava* L. | Guava | 3.3 | 3.3 | 0.0 | 0.0 | 0.0 |  | 8.0 | 7.5 | 0.0 | 0.5 | 0.0 |
| *Ocimum basilicum* L. | Sweet basil | 1.0 | 1.0 | 0.0 | 0.0 | 0.0 |  | 4.8 | 4.5 | 0.0 | 0.2 | 0.0 |
| *Persea americana* Mill. | Avocado | 0.3 | 0.3 | 0.0 | 0.0 | 0.0 |  | 3.4 | 0.9 | 0.5 | 1.8 | 0.2 |
| *Anacardium occidentale* L. | Cashew | 2.3 | 1.3 | 0.3 | 0.7 | 0.0 |  | 1.4 | 0.7 | 0.7 | 0.0 | 0.0 |
| *Colocasia esculenta*(L.) Schott | Cocoyam | 2.3 | 2.0 | 0.0 | 0.3 | 0.0 |  | 1.1 | 0.7 | 0.0 | 0.5 | 0.0 |
| *Citrus limon* (L.) Osbeck | Lemon | 0.7 | 0.0 | 0.3 | 0.3 | 0.0 |  | 2.5 | 1.6 | 0.5 | 0.5 | 0.0 |
| *Launaea taraxacifolia* (Willd.) Amin ex C. Jeffrey | African lettuce | 0.0 | 0.0 | 0.0 | 0.0 | 0.0 |  | 2.5 | 2.5 | 0.0 | 0.0 | 0.0 |
| *Sorghum bicolor* (L.) Moench | Sorghum | 1.3 | 1.3 | 0.0 | 0.0 | 0.0 |  | 1.1 | 0.5 | 0.0 | 0.7 | 0.0 |
| *Dialium guineense* Willd. | Velvet tamarind | 0.0 | 0.0 | 0.0 | 0.0 | 0.0 |  | 2.3 | 2.0 | 0.0 | 0.2 | 0.0 |
| *Chrysophyllum albidum* G. Don | Yellow mombin | 0.7 | 0.7 | 0.0 | 0.0 | 0.0 |  | 1.6 | 1.1 | 0.2 | 0.2 | 0.0 |
| *Cucurbita spp* | pumpkin | 0.0 | 0.0 | 0.0 | 0.0 | 0.0 |  | 1.6 | 0.5 | 0.0 | 1.1 | 0.0 |
| *Cajanus cajan* (L.) Millsp. | pigeon pea | 0.7 | 0.7 | 0.0 | 0.0 | 0.0 |  | 0.9 | 0.5 | 0.0 | 0.5 | 0.0 |
| *Artocarpus altilis* (Parkinson ex F.A.Zorn) Fosberg | Breadfruit | 0.0 | 0.0 | 0.0 | 0.0 | 0.0 |  | 1.4 | 0.9 | 0.0 | 0.5 | 0.0 |
| *Crassocephalum rubens* (Juss. ex Jacq.) S. Moore | Yoruban bologi | 0.3 | 0.3 | 0.0 | 0.0 | 0.0 |  | 0.9 | 0.5 | 0.0 | 0.5 | 0.0 |
| *Vigna subterranea* (L.) Verdc. | Bambara nut | 1.0 | 1.0 | 0.0 | 0.0 | 0.0 |  | 0.2 | 0.2 | 0.0 | 0.0 | 0.0 |
| *Citrus reticulata* Blanco | Mandarin | 0.0 | 0.0 | 0.0 | 0.0 | 0.0 |  | 1.1 | 0.5 | 0.0 | 0.7 | 0.0 |
| *Cocos nucifera* L. | Coconut | 0.0 | 0.0 | 0.0 | 0.0 | 0.0 |  | 0.9 | 0.5 | 0.0 | 0.5 | 0.0 |
| Talinum fruticosum (L.) Juss. | Waterleaf | 0.0 | 0.0 | 0.0 | 0.0 | 0.0 |  | 0.9 | 0.9 | 0.0 | 0.0 | 0.0 |
| *Cola nitida* (Vent.) Schott & Endl. | Bitter cola | 0.0 | 0.0 | 0.0 | 0.0 | 0.0 |  | 0.7 | 0.2 | 0.0 | 0.5 | 0.0 |
| Milicia excelsa | Iroko | 0.0 | 0.0 | 0.0 | 0.0 | 0.0 |  | 0.7 | 0.7 | 0.0 | 0.0 | 0.0 |
| *Saccharum officinarum* L. | Sugar cane | 0.3 | 0.0 | 0.3 | 0.0 | 0.0 |  | 0.2 | 0.0 | 0.0 | 0.2 | 0.0 |
| *Stachytarpheta indica* (L.) Vahl | Indian snakeweed | 0.3 | 0.3 | 0.0 | 0.0 | 0.0 |  | 0.2 | 0.2 | 0.0 | 0.0 | 0.0 |
| *Cymbopogon citratus*(DC.) Stapf | Lemongrass | 0.0 | 0.0 | 0.0 | 0.0 | 0.0 |  | 0.5 | 0.2 | 0.0 | 0.2 | 0.0 |
| Manihot carthaginensis subsp. glaziovii | Ceara rubber tree | 0.0 | 0.0 | 0.0 | 0.0 | 0.0 |  | 0.5 | 0.2 | 0.0 | 0.2 | 0.0 |
| Saba senegalensis (A.DC.) Pichon | Senegal saba | 0.0 | 0.0 | 0.0 | 0.0 | 0.0 |  | 0.5 | 0.0 | 0.2 | 0.2 | 0.0 |
| *Annona muricata* L. | Coursop | 0.3 | 0.0 | 0.0 | 0.3 | 0.0 |  | 0.0 | 0.0 | 0.0 | 0.0 | 0.0 |
| *Citrus maxima* (Burm.) Merr. | Pomelo | 0.3 | 0.0 | 0.3 | 0.0 | 0.0 |  | 0.0 | 0.0 | 0.0 | 0.0 | 0.0 |
| Parkia biglobosa (Jacq.) G.Don | African locust bean | 0.3 | 0.0 | 0.3 | 0.0 | 0.0 |  | 0.0 | 0.0 | 0.0 | 0.0 | 0.0 |
| *Solanum aethiopicum* L. | Eggplant | 0.3 | 0.0 | 0.3 | 0.0 | 0.0 |  | 0.0 | 0.0 | 0.0 | 0.0 | 0.0 |
| *Adansonia digitata* L. | Baobab | 0.0 | 0.0 | 0.0 | 0.0 | 0.0 |  | 0.2 | 0.2 | 0.0 | 0.0 | 0.0 |
| *Annona squamosa* L. | Sugar apple | 0.0 | 0.0 | 0.0 | 0.0 | 0.0 |  | 0.2 | 0.0 | 0.0 | 0.2 | 0.0 |
| *Cleome gynandra L.* | Spiderplant | 0.0 | 0.0 | 0.0 | 0.0 | 0.0 |  | 0.2 | 0.2 | 0.0 | 0.0 | 0.0 |
| *Astraea lobata* (L.) Klotzsch | Lilac bush | 0.0 | 0.0 | 0.0 | 0.0 | 0.0 |  | 0.2 | 0.2 | 0.0 | 0.0 | 0.0 |
| *Dioscorea dumetorum* (Kunth) Pax | Better yam | 0.0 | 0.0 | 0.0 | 0.0 | 0.0 |  | 0.2 | 0.0 | 0.0 | 0.2 | 0.0 |
| Ficuspolita Vahl | Heart-leaved fig | 0.0 | 0.0 | 0.0 | 0.0 | 0.0 |  | 0.2 | 0.2 | 0.0 | 0.0 | 0.0 |
| *Cnidoscolus aconitifolius* (Mill.) I.M Johnst. | Chaya | 0.0 | 0.0 | 0.0 | 0.0 | 0.0 |  | 0.2 | 0.2 | 0.0 | 0.0 | 0.0 |
| *Lens culinaris* Medik. | Lentil | 0.0 | 0.0 | 0.0 | 0.0 | 0.0 |  | 0.2 | 0.0 | 0.0 | 0.2 | 0.0 |
| *Struchium sparganophorum* (L.) Kuntze |  | 0.0 | 0.0 | 0.0 | 0.0 | 0.0 |  | 0.2 | 0.2 | 0.0 | 0.0 | 0.0 |
| *Synsepalum dulcificum* (Schumach. & Thonn.) Daniell | Miracle fruit | 0.0 | 0.0 | 0.0 | 0.0 | 0.0 |  | 0.2 | 0.2 | 0.0 | 0.0 | 0.0 |
| *Theobroma cacao* L. | Cocoa | 0.0 | 0.0 | 0.0 | 0.0 | 0.0 |  | 0.2 | 0.2 | 0.0 | 0.0 | 0.0 |
