# Supplementary material for: On-Farm Diversity and Market Participation Are Positively Associated with Dietary Diversity of Rural Mothers in Southern Benin, West Africa
Source: PLoS One. 2016 Sep 8;11(9):e0162535. doi: 10.1371/journal.pone.0162535 (PMC5015832; doi:10.1371/journal.pone.0162535)
Supplement: S3 Table — (DOCX) [file pone.0162535.s003.docx]

Table S3. Results of a system of simultaneous equations modeling OFD, MD and DD as outcome variables including an interaction term between OFD and the rural market-shed

| Variable | On Farm Diversity | |  | Market Diversity | |  | Dietary Diversity |  |  |
| --- | --- | --- | --- | --- | --- | --- | --- | --- | --- |
| Constant | 6.387 | ** | | | 16.845 |  | 3.734 |  | |
| OFD |  |  | | |  |  | 0.048 | * | |
| OFD×Rural market-shed |  |  | | |  |  | -0.090 |  | |
| MD |  |  | | |  |  | 0.029 | * | |
| Landholdings | 0.126 | *** | | | -0.271 |  |  |  | |
| Square of landholdings | -0.006 |  | | | -0.001 |  |  |  | |
| Socioeconomic Index | -0.019 |  | | | 1.054 | ** |  |  | |
| Urban market-shed | 0.538 | *** | | | -0.770 |  | -0.265 |  | |
| Semi-urban market-shed | -0.088 |  | | | -2.680 |  | -0.399 |  | |
| Travel time | 0.016 | *** | | | -0.195 | *** | 0.009 |  | |
| Square of travel time | -0.000 | *** | | | 0.001 | ** | -0.000 |  | |
| No. non-agricultural income sources –Father | 0.015 |  | | | 0.277 |  | -0.018 |  | |
| Agriculture rated very important income source-Father | 0.491 | *** | | | -0.293 |  | -0.008 |  | |
| Agriculture rated important income source-Father | 0.477 | *** | | | -0.225 |  | -0.030 |  | |
| No. non-agricultural income sources – Mother | 0.154 | *** | | | 0.236 |  | 0.003 |  | |
| Agriculture rated very important income source-Mother | 0.354 | *** | | | -2.587 | *** | 0.059 |  | |
| Agriculture rated important income source-Mother | 0.378 | *** | | | -0.531 |  | -0.013 |  | |
| Mother age | -0.007 | * | | | 0.094 | * | -0.002 |  | |
| Mother education | -0.005 |  | | | 0.313 | ** | 0.004 |  | |
| Mother ethnicity (aizo) | 0.056 |  | | | -2.985 | *** | 0.072 |  | |
| Family size | 0.032 | *** | | | 0.072 |  | -0.001 |  | |
| Temperature range | -1.223 |  | | | -5.222 |  | -0.382 |  | |
| Coefficient of variation precipitation | -0.035 |  | | | 0.706 |  | -0.057 |  | |
| Precipitation range | 0.001 |  | | | -0.058 |  | 0.005 |  | |
| Season | -0.518 | *** | | | -1.178 | * | 0.176 | * | |

^1^N=878, Significance at the .05, .01, .001 level indicated by *, **, *** respectively for a two-tail t-test
