# Supplementary material for: On-Farm Diversity and Market Participation Are Positively Associated with Dietary Diversity of Rural Mothers in Southern Benin, West Africa
Source: PLoS One. 2016 Sep 8;11(9):e0162535. doi: 10.1371/journal.pone.0162535 (PMC5015832; doi:10.1371/journal.pone.0162535)
Supplement: S4 Table — (DOCX) [file pone.0162535.s004.docx]

Table S4. Results of a system of simultaneous equations modeling OFD, MD and Minimum Dietary Diversity – Women^1,2^ as outcome variables

| Variable | On Farm Diversity | |  | Market Diversity | |  | MDD_W^3^ |  |  |
| --- | --- | --- | --- | --- | --- | --- | --- | --- | --- |
| Constant | 29.904 |  | | | 16.845 |  | 3.004 |  | |
| OFD |  |  | | |  |  | 0.216 | ** | |
| MD |  |  | | |  |  | 0.153 | * | |
| Landholdings | 0.778 | *** | | | -0.271 |  |  |  | |
| Square of landholdings | -0.037 | *** | | | -0.001 |  |  |  | |
| Socioeconomic Index | -0.144 |  | | | 1.054 | *** |  |  | |
| Urban market-shed | 2.741 | *** | | | -0.770 |  | 0.346 |  | |
| Semi-urban market-shed | -0.702 |  | | | -2.680 |  | 0.065 |  | |
| Travel time | 0.074 | *** | | | -0.195 | *** | 0.031 |  | |
| Square of travel time | -0.001 | * | | | 0.001 | ** | 0.000 |  | |
| No. non-agricultural income sources –Father | 0.301 | *** | | | 0.277 |  | -0.060 |  | |
| Agriculture rated very important income source-Father | 1.850 | *** | | | -0.293 |  | -0.323 |  | |
| Agriculture rated important income source-Mother | 1.627 | *** | | | -0.225 |  | -0.417 |  | |
| No. non-agricultural income sources – Father | 0.654 | *** | | | 0.236 |  | -0.216 | * | |
| Agriculture rated very important income source-Mother | 1.916 | *** | | | -2.587 | *** | 0.064 |  | |
| Agriculture rated important income source-Mother | 2.113 | *** | | | -0.531 |  | -0.253 |  | |
| Mother age | -0.027 |  | | | 0.094 | * | 0.000 |  | |
| Mother education | 0.012 |  | | | 0.313 | *** | 0.003 |  | |
| Mother ethnicity (aizo) | 0.393 |  | | | -2.985 | *** | 0.459 |  | |
| Family size | 0.142 | ** | | | 0.072 |  | -0.021 |  | |
| Temperature range | -6.036 | *** | | | -5.222 |  | -0.578 |  | |
| Coefficient of variation precipitation | -0.201 |  | | | 0.706 |  | -0.191 |  | |
| Precipitation range | 0.009 |  | | | -0.058 |  | 0.017 |  | |
| Season | -2.395 | *** | | | -1.178 | * | 1.304 | *** | |

^1^N=878, Significance at the .05, .01, .001 level indicated by *, **, *** respectively for a two-tail t-test

^2^Estimates were obtained using maximum likelihood estimator [70] assuming OFD and MD as continuous endogenous variables, and MDD-W as dichotomous:

$MDD-W_{i}^{*}=\mathbf{x}'_{\mathbf{i}}\mathbf{\omega}+\delta OFD_{i}+\zeta MD_{i}+e_{i}$ (Eq. a1)

 (Eq. a2)

 (Eq. a3)

with MDD-W_i_ = 1 if MDD-W^*^_i_ > 0, MDD-W_i_ = 1 if MDD-W^*^_i_ ≤ 0.

^3^Minimum Dietary Diversity – Women (dummy of whether mother had an intake of at least 15grams for each for 5 food groups or more out of 10).

**Reference**

70. Amemiya T. The estimation of a simultaneous equation generalized probit model. Econometrica. 1978; 46: 1193–1205.
