# Supplementary material for: On-Farm Diversity and Market Participation Are Positively Associated with Dietary Diversity of Rural Mothers in Southern Benin, West Africa
Source: PLoS One. 2016 Sep 8;11(9):e0162535. doi: 10.1371/journal.pone.0162535 (PMC5015832; doi:10.1371/journal.pone.0162535)
Supplement: S1 Text — (DOCX) [file pone.0162535.s005.docx]

**S1 Text**

Sample size determination

Sample size was determined using a standard formula [49]:

N= [Z² x p(1-p) /m²] x DE x 1/(1-λ)

where: N = estimated sample size in each department; Z = level of confidence at 95% (value type of 1.96); p = estimated prevalence of mothers’ underweight in each Department according to available DHS data; m = margin of error at 5%; DE = design effect fixed at 1.4; and λ = refusal rate fixed at 10%. The largest size obtained from one départment (n = 238) was multiplied by two to obtain the final sample size (N = 476).
